# *Trypanosoma cruzi* Parasite Burdens of Several Triatomine Species in Colombia

**DOI:** 10.3390/tropicalmed7120445

**Published:** 2022-12-19

**Authors:** Natalia Velásquez-Ortiz, Carolina Hernández, Omar Cantillo-Barraza, Nathalia Ballesteros, Lissa Cruz-Saavedra, Giovanny Herrera, Luz Stella Buitrago, Hugo Soto, Manuel Medina, Jatney Palacio, Marina Stella González, Andrés Cuervo, Gustavo Vallejo, Liliana Zuleta Dueñas, Plutarco Urbano, Marina Muñoz, Juan David Ramírez

**Affiliations:** 1 Centro de Investigaciones en Microbiología y Biotecnología-UR (CIMBIUR), Facultad de Ciencias Naturales, Universidad del Rosario, Bogotá, Colombia; 2 Centro de Tecnología en Salud (CETESA), Innovaseq SAS, Bogotá, Colombia; 3 Grupo BCEI, Universidad de Antioquia, Medellín, Colombia; 4 Laboratorio de Salud Pública del Meta, Villavicencio, Colombia; 5 Laboratorio de Salud Pública del Cesar, Valledupar, Colombia; 6 Programa de Control de ETV, Secretaría de Salud de Boyacá, Tunja, Colombia; 7 Laboratorio Departamental de Salud Pública del Departamento del Chocó, Quibdó, Colombia; 8 Secretaría Departamental de Salud de Arauca, Arauca, Colombia; 9 Laboratorio de Investigaciones en Parasitología Tropical, Facultad de Ciencias, Universidad del Tolima, Ibagué, Colombia; 10 Grupo de Vigilancia en Salud Pública, Secretaría de Salud de Casanare, Yopal, Colombia; 11 Grupo de Investigaciones Biológicas de la Orinoquia, Universidad Internacional del Trópico Americano (Unitrópico), Yopal, Colombia; 12 Molecular Microbiology Laboratory, Department of Pathology, Molecular and Cell-Based Medicine, Icahn School of Medicine at Mount Sinai, New York City, NY, USA

**Keywords:** *Trypanosoma cruzi*, parasite load, triatomines, Colombia, qPCR

## Abstract

*Trypanosoma cruzi*, the causal agent of Chagas disease, is mainly transmitted by insects of the Triatominae subfamily. In Colombia, there are 26 triatomine species, and 16 of them are naturally infected with the parasite. The parasite loads of naturally infected vectors can be significant in targeting specific species that can affect the epidemiology of the disease. Studying their ecology and behavior is vital to understand their role in *T. cruzi* transmission dynamics. We evaluated the parasite loads of 182 field-collected triatomines corresponding to 10 species in 13 departments across Colombia. We standardized a methodology to quantify *T. cruzi* DNA in these insects. We obtained a LOD (limit of detection) of 3.05 p-eq/mL. The 82% of triatomines we evaluated were positive for *T. cruzi* infection, with loads ranging from hundreds to millions of equivalent parasites per milliliter. *Panstrongylus geniculatus*, *Rhodnius prolixus*, and *Triatoma dimidiata* were the species with the highest loads of *T. cruzi*; however, other species whose role as vectors is still unknown were also found with high loads of parasites. Our results suggest the relevance of secondary species for *T. cruzi* transmission in Colombia. We hope our data can help improve entomological surveillance and vector control programs in the country and the region.

## 1. Introduction

Chagas disease (CD) is a neglected zoonosis with a high impact in Latin America [1]. It is caused by the parasite *Trypanosoma cruzi* [2], whose transmission is mediated by insects of the subfamily Triatominae (Hemiptera) [1]. *Trypanosoma cruzi* is subdivided into seven discrete typing units (DTUs): TcI (the most diverse and widely distributed in the Americas) to TcVI, and TcBat (associated with bats) [3]. Estimates indicate six million people infected and 30,000 annual vector-borne cases [4]. The endemic countries for CD have implemented control programs and cooperation between them to make efforts that allow the interruption of *T. cruzi* vector-borne transmission. However, there are more than a hundred native and non-native vector species in the Americas, some of them that can invade or reinfest. Therefore, it is difficult to reach the complete interruption of *T. cruzi* transmission by targeting its vectors, but knowing their behavior is vital for CD control [5].

The Triatominae are classified into 5 tribes and 15 genera [6], but three are mainly related to transmission to humans: *Triatoma* (Laporte, 1832), *Rhodnius* (Stål, 1859) and *Panstrongylus* (Berg, 1879) [6]. The parasite triatomine feces transmit the parasite, which contains the infective forms of *T. cruzi* (metacyclic trypomastigotes), when they feed on the mammalian host and defecate later [2]. In Colombia, 26 triatomine species have been reported, but only 16 are considered to be naturally infected with *T. cruzi* [7,8]. *Rhodnius prolixus* (Stål, 1859) and *Triatoma dimidiata* (Latreille, 1811) are the main species and primary vectors transmitting the parasite [7,9]. However, in recent years, some species considered secondary vectors (*Panstrongylus geniculatus* (Latreille, 1811), *Triatoma maculata* (Erichson, 1848), *Rhodnius pallescens* (Barber, 1932), and *Triatoma venosa* (Stål, 1872)) were found to feed on human blood and be infected with sylvatic DTUs [10,11].

In the last years, many triatomine species have become important for *T. cruzi* transmission due to the elimination of the primary vectors (e.g., *R. prolixus*) and reports of oral outbreaks of CD in endemic and non-endemic regions [7,10,12,13]. This situation implies some species could reach human dwellings and colonize because these structures can be adequate niches for triatomine species, increasing the risk of CD transmission [14]. Moreover, few studies have recently evaluated the parasitic load in vectors using molecular methods such as quantitative PCR (qPCR) instead of classic microscopy. One of them found 1.29 × 10^9^ parasites in the Chilean vector *Mepraia spinolai* (Porter, 1934) [15]. The other was made for *T. dimidiata* in Boyacá, finding loads as large as 1.46 × 10^9^ parasite equivalents [9]. These values indicate the high quantity of parasites that some species could harbor, and that this parameter should be more relevant, considering the importance of the parasite inoculum for disease development [12].

Currently, more studies aiming to evaluate the parasitic load of naturally infected vectors are needed, which can be important for vector control programs to help target certain species that can influence the epidemiology of the disease. There is evidence of behavioral changes in the triatomines according to their infection status, such as a reduction in defecation time, improvement in host detection, a higher number of bites, reduction in feeding frequency, locomotor behavior, and fertility, among others [16,17,18,19,20,21,22,23,24]. Thus, there is an imminent risk for humans, and it is essential to track vector species and understand their ecology, behavior, and role in *T. cruzi* transmission. Hence, considering the relevance of the parasite inoculum for CD development, the vast number of triatomine species present in Colombia, and the need to prioritize some species for building efficient vector control programs. We aimed to describe a methodology to quantify parasite loads of *T. cruzi* and its ecoepidemiological implications in ten triatomine species collected in the field across 13 departments of Colombia that are endemic to CD.

## 2. Materials and Methods

### 2.1. Sample Information and T. cruzi Detection

**Figure 1:**
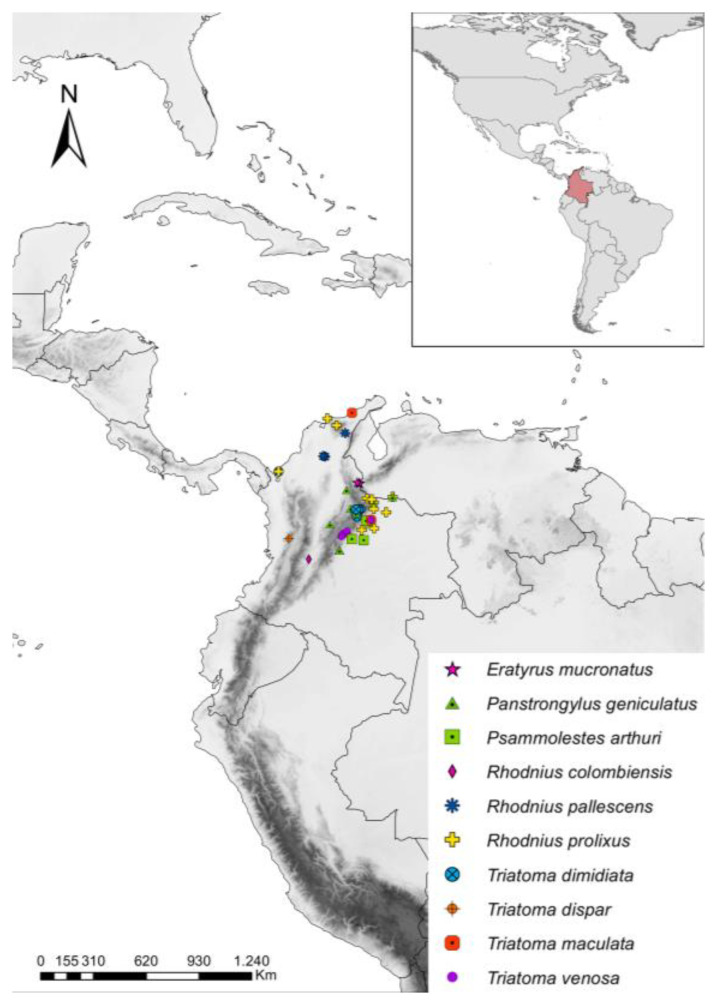
Distribution of triatomines per species collected in the field across 13 departments of Colombia. The map was made using ArcGIS (Software) v10.2 (ESRI, 2014).

We selected 275 samples (extracted DNA and specimens) from triatomines that were field-collected between 2014 and 2022. Of these, we randomly selected 182 samples explicitly collected in Colombia. We standardized a quantitative real-time PCR (qPCR) that was employed for estimating parasitic loads on 182 triatomine DNA samples from the following species: *Eratyrus mucronatus* (Stål, 1859) (4)*, Panstrongylus geniculatus* (23)*, Psammolestes arthuri* (Pinto, 1926) (25)*, Rhodnius colombiensis* (Moreno, Jurberg and Galvão, 1999) (13)*, Rhodnius pallescens* (19)*, Rhodnius prolixus* (37)*, Triatoma dimidiata* (30)*, Triatoma maculata* (2), *Triatoma dispar* (Lent, 1950) (2), and *Triatoma venosa* (27), collected in the field from 13 departments in Colombia (Antioquia, Arauca, Bolívar, Boyacá, Casanare, Cesar, Chocó, Guajira, Magdalena, Meta, Norte de Santander, Santander, and Tolima) (Figure 1). The DNA of the insects was extracted from the gut and rectal ampulla using a DNeasy Blood and Tissue kit (Qiagen, Berlin, Germany). The specimens were identified using the taxonomy key of Lent and Wygodzinsky, except *T. venosa* specimens that were identified using the TriatoDex App [25]. Of the 182 samples, 97.8% were adults, and only 2.2% percent (4 individuals) were nymphs belonging to *T. dimidiata* (Table S1).

### 2.2. T. cruzi Quantification and qPCR Standardization

The qPCR protocol was first evaluated and validated for *T. cruzi* detection in blood samples from Chagas disease patients [26]. Here, we standardized a qPCR protocol to obtain the parasite load from triatomines collected in the field. For this, we used *T. cruzi* MHOM/CO/04/MG strain (TcI) metacyclic trypomastigotes, as reported previously [27], that were isolated and quantified to obtain 10^11^ parasites per mL. We took the supernatant from the culture flasks, verifying that there were an abundant number of trypomastigotes, and put them in a 15 mL falcon tube. The parasites were concentrated by centrifugation (5 min at 1500 rpm) and then were washed with ultra-cold PBS, and the pellet was resuspended. A 20 µL aliquot was obtained at the end. The parasites were quantified in the Neubauer counting chamber using 10 µL of the previously aliquoted suspension. Serial dilutions of 1/100 were made until it was possible to count the parasites per quadrant. Then, we calculated the number of parasites per mL. After that, we took a 1 mL aliquot from the initial parasite’s suspension (no dilutions), centrifuged it 5 min at 1500 rpm, removed 500 mL of supernatant, and resuspended the pellet (parasites) in the remaining 500 µL.

For the DNA extraction process, we added the 500 µL resuspended solution with trypomastigotes to the abdomen section of colony non-infected adult specimens of *R. prolixus* prior to the disruption step. This allowed shredding both parasites and insect cells using lysis buffer and FastPrep lysis beads and matrix tubes. The DNA extraction procedures were performed using the Dneasy Blood and Tissue Kit (Qiagen, Hilden, Germany). Then, with the DNA obtained, we prepared the parasite standard curve through serial dilutions from 10^11^ to 10^−1^ parasites per mL and stored them at −20 °C. The qPCR was run with the standard curve (serial dilutions) placed by duplicate in every qPCR plate along with 5 µL of samples’ DNA to obtain the parasite load.

Reaction reagents for the qPCR were a TaqMan Fast Advanced Master Mix 2× (Roche Diagnostics GmbH, Mannheim, Germany); water; and the primers cruzi1 (0.75 μM) (5′-AST CGG CTG ATC GTT TTC-3′), cruzi2 (0.75 μM) (5′-AAT TCC TCC AAG CAG CGG ATA-3′), and a cruzi3 probe (0.05 μM) (FAM-CAC ACA CTG GAC ACC AA-NFQ-MGB), targeting the satellite DNA of *Trypanosoma cruzi* (166 bp), as previously reported [28]. Reaction conditions were as follows: (i) first step: 50 °C for 2 min; (ii) second step: 95 °C for 10 min; and (iii) third step (40 cycles): 95 °C for 15 s, and 58 °C for 1 min. The nuclear gene 28S rDNA was used as internal amplification control by conventional PCR using the primers D2F (5′-GCGAGTCGTGTTGCTTGA TAGTGCAG-3′) and D2R (5′-TTGGTCCGTGTTTCAAGACGGG-3′) following the conditions described previously [29]. The quantity of parasites per sample was calculated automatically by the Real-Time Software Quant Studio Design and Analysis (Applied Biosystems) using the linear regression equation obtained from the standard curve.

### 2.3. Standard Calibration, Limit of Detection (LOD), and Parasite Burdens

As mentioned previously, the curve was made by serial dilutions from the initial stock of 10^11^ metacyclic trypomastigotes (MG strain) and diluted to 10^−1^ parasites per mL using DNA from the intestinal content of non-infected reared *R. prolixus*, following the conditions previously described [30]. Each dilution was tested in duplicate, and the LOD was calculated using the Probit regression analysis and the formula LOD = |3.3(Sy/S)|, where Sy is the standard error of the regression and S is the slope. The information from the standard curve was used to extrapolate the Ct of the unknown samples (DNA samples from the different species) and calculate the estimated parasitic load per specimen. Parasite load boxplot was made with Rstudio v.4.1.2 with the package ggplot2. The values on the Y axis were transformed to Log10 scale to enhance visualization of the data.

### 2.4. Parasite Genotyping

Parasite genotyping was made through conventional PCR by amplifying the spliced leader intergenic region of the miniexon gene (SL-IR) (TcI: 350 bp, TcII-TcVI: 300 bp) for the qPCR-positive samples. Reaction reagents used were the following: Go Taq Green Master Mix 1×; water; and primers TCC (1.25 nM) (50-CCC CCC TCC CAG GCC ACA CTG-30), TC1 (1.25 nM) (5′-GTG TCC GCC ACC TCC TTC GGG CC-3′), and TC2 (1.25 nM) (5′-CCT GCA GGC ACA CGT GTG TGT G-3′) [31]. PCR products were analyzed by electrophoresis on 2% agarose gels stained with SYBR Safe™ (Invitrogen, Carlsbad, CA, USA), generating a product with a length of 300 bp for TcII and 350 bp for TcI.

### 2.5. Statistical Analysis

We produced a Kolmogorov–Smirnov normality test for the 182 samples. Then, a non-parametric Wilcoxon test and a Kruskal–Wallis test were produced to compare the parasite load values (converted to Log10 scale) per department and triatomine species. Post hoc tests (Dunn tests with Bonferroni method adjust) were produced to correct the previous tests per sample size. All the tests were run in Rstudio v4.1.2 and the package Rcmdr v2.7.2.

## 3. Results

### 3.1. Standard Curve and LOD

**Figure 2:**
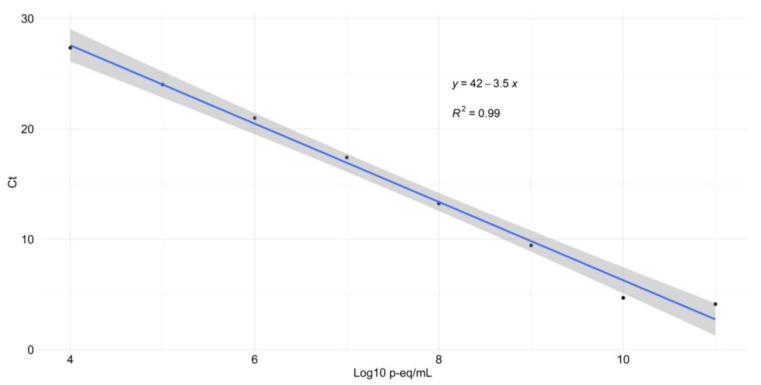
Linear regression plot of the standard curve. *X*-axis values for equivalent parasites were transformed to Log10 scale.

The curve was made using a TcI strain of *T. cruzi* and diluted with non-infected intestinal content of *R. prolixus* specimens in duplicate. The average values of the equation parameters showed an R^2^ value of 0.990, an efficiency of 91.4%, a slope of −3.546, a Y-intercept of 41.753, and a regression error of 0.926 (Figure 2). The Probit analysis showed a LOD value of 3.05 p-eq/mL (CI 95%; −7.02–13.13).

### 3.2. T. cruzi Infection, Parasite Loads, and DTUs

**Figure 3:**
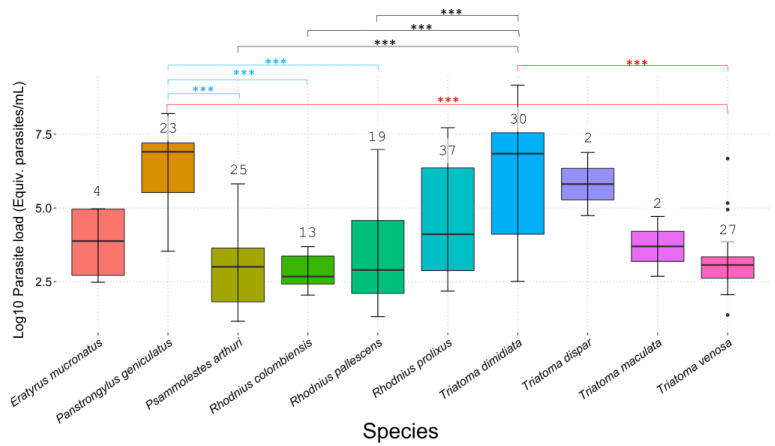
Boxplot of parasitic loads per species. *Y*-axis was transformed to Log10 scale for better visualization of the data. The number above the boxes is the number of samples per species. Asterisks (***) represent statistically significant comparisons (*p* < 0.0001). In blue, *P. geniculatus* with *Ps. arthuri*, *R. colombiensis,* and *R. pallescens*; in black, *T. dimidiata* with *Ps. arthuri*, *R. colombiensis*, and *R. pallescens*; and in red, *T. venosa* with *P. geniculatus* and *T. dimidiata*.

**Figure 4:**
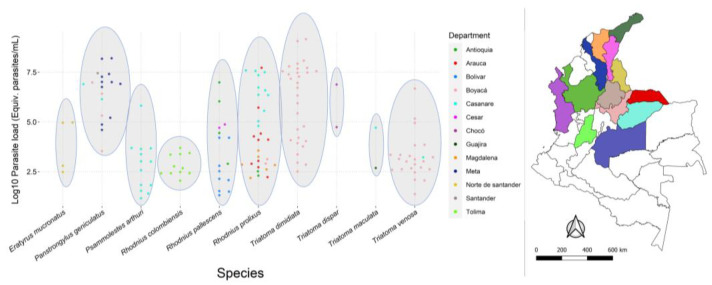
Beeswarm plot of the parasite loads per species and their distribution per department. Blue ovals encircle the samples per species. The map on the right shows the departments geographic location in Colombia and their corresponding colors with the dots on the left. The map was made with QGIS v3.22.

Of the 182 triatomines evaluated, 82.4% (150/182) were positive for *T. cruzi* infection, and the rates per species were the following: *E. mucronatus* (4/4; 100%)*, P. geniculatus* (18/23; 78%)*, Ps. arthuri* (13/25; 52%)*, R. colombiensis* (11/13; 85%)*, R. pallescens* (15/19; 79%)*, R. prolixus* (31/37; 84%), *T. dimidiata* (30/30; 100%)*, T. dispar* (2/2; 100%)*, T. maculata* (2/2; 100%), and *T. venosa* (24/27; 89%) (Figure S1). For the positive samples, *P. geniculatus*, *R. prolixus*, and *T. dimidiata* were the species with the highest loads of *T. cruzi* (Figure 3), and *P. geniculatus*, *R. pallescens*, and *R. prolixus* were the only species collected in more than two departments (Figure 4, Figure S2). For better visualization of the data, the parasite loads were transformed to Log10 values, with 1 being an order of 10, 2 of 100, 3 of 1000, 4 of 1 × 10^4^, 5 of 1 × 10^5^, 6 of 1 × 10^6^, 7 of 1 × 10^7^, and 8 of 1 × 10^8^.

Regarding the DTUS, only TcI was detected in 79 of the 182 samples and TcII in 1 sample of *P. geniculatus*; 66 samples were unable to be genotyped by conventional PCR (NA) (Table S2).

### 3.3. Statistical Analysis

The data obtained did not fit the normal distribution (*p* < 0.05), and therefore we proceeded to evaluate different variable comparisons using non-parametric tests. A Wilcoxon test of the Log10 parasite load values was statistically significant (*p* < 0.05), and the Kruskal–Wallis tests comparing parasite loads per department and per species were both statistically significant (*p* = 0.00007796, *p* = 2.85 × 10^−10^, respectively). Then, a post hoc Dunn test with Bonferroni’s correction for both Kruskal–Wallis tests were made. For the comparison per department, four paired comparisons were significant with the adjusted *p* value (*p* < 0.05): Bolivar–Boyacá, Bolivar–Meta, Magdalena–Meta, and Meta–Tolima. Per species, eight paired comparisons were statistically significant (*p* < 0.05): *P. geniculatus* with *Ps. arthuri*, *R. colombiensis,* and *R. pallescens*; *T. dimidiata* with *Ps. arthuri*, *R. colombiensis*, and *R. pallescens*; and *T. venosa* with *P. geniculatus* and *T. dimidiata* (Figure 3).

## 4. Discussion

Vector transmission of *T. cruzi* is still the most important transmission mechanism causing many infections annually [5]. In the last years, parasite load quantification has been evaluated using standard curves but mainly using human samples allowing the improvement of the detection of the parasite at the clinical level [32,33] using the qPCR assay targeting the tandem repeat satellite DNA of *T. cruzi*, proven to be an excellent method for parasite detection [34]. This tool could improve the analysis capacity, decision-making, management, and evaluation of the entomological surveillance programs in endemic zones, mainly because it can help to identify risk factors in potential transmission areas, track the vectors’ infection, and target epidemiologically important species [35].

We implemented and standardized for the first time a qPCR to quantify the parasite loads of triatomine vectors, running a standard curve using metacyclic trypomastigotes of *T. cruzi* and colony non-infected specimens of *R. prolixus* as matrix. For the LOD of the standard curve, we found a value of 3.05 p-eq/mL, interpreted as the smallest number of parasites that the qPCR can detect, with an interval of −7.02 to 13.13 p-eq/mL. This was calculated with a 95% confidence interval, which along with the R^2^ value of 0.990 and the efficiency of 91.4% (Figure 2) provided us with good reliability, analytical sensitivity, and good qPCR performance for *T. cruzi* quantification. A similar study in Brazil for field-collected triatomines found high sensitivity of the qPCR with a LOD of 0.41 parasites in the gut [36]. Accordingly, with the methodology implemented, we obtained 150 of 182 positive insects for *T. cruzi* with parasite loads ranging from hundreds to millions of equivalent parasites per milliliter, with significant differences among certain species that will subsequently be discussed. Our results showed that our qPCR was highly sensitive in evaluating the parasite load, an important parameter to further evaluate the vector capacity of triatomines epidemiologically relevant in CD endemic zones.

Regarding the infection and parasite loads, as expected, we found *P. geniculatus*, *R. prolixus*, and *T. dimidiata* were the species with the highest quantities of parasites (Figure 3). This implies Colombia has excelling vectors for Chagas disease, even though some other species ranked between a hundred and a thousand parasites such as *R. pallescens*, *E. mucronatus, T. maculata, T. venosa, Ps. arthuri,* and *R. colombiensis* (Figure 3). Moreover, *P. geniculatus*, *R. pallescens,* and *R. prolixus* were the species collected in more departments, showing their broad distribution in the country (Figure 4 and Figure S2). However, the ranges some triatomine species have is interesting, even though their role as vectors is still unknown, such as *Psammolestes arthuri* [8], and other species considered secondary vectors such as *E. mucronatus* and *R. pallescens* [7,10,37,38,39]. In the case of *T. venosa*, it presented many parasites in a narrower range. Still, in recent years, it is becoming important as a secondary vector due to its domiciliation along *T. dimidiata* following the elimination of *R. prolixus* in Boyacá, Colombia [9,11]. The above suggests that the secondary vectors could have similar parasite loads to those of the primary vectors and play an essential role in the parasite’s transmission cycle. Therefore, along with the evidence of domiciliation processes of some of these species, we highlight the need to include them in the vector control programs to evaluate their vector capacity and more aspects of their biology and ecology.

Considering the above, we found there was statistically significant differences among the parasite load values (*p* < 0.05). Thus, we performed comparisons per department and species that also were statistically significant (*p* < 0.05). The four significant comparisons were the following: Bolivar–Boyacá, Bolivar–Meta, Magdalena–Meta, and Meta–Tolima. For Caribbean departments such as Bolivar and Magdalena, the parasite’s transmission is associated with secondary vectors with no evidence of domiciliation but an active *T. cruzi* transmission [40,41], and the parasite loads might be associated with the presence of reservoirs such as *Didelphis marsupialis* and dogs, which have high infection rates and seroprevalence [40,41]. In Colombia’s Caribbean, the main vector species are *R. pallescens* and *T. maculata*. The first one is associated with the palm tree *Attalea butyracea* [41], and the second was found to mediate the peridomestic transmission [40]. Here, for Bolivar and Magdalena, we found *R. pallescens* and *R. prolixus*, respectively. However, the ecoepidemiological situation for *R. prolixus* in Magdalena (specifically Sierra Nevada de Santa Marta) is different because it is found intradomiciliary, with high infection rates and feeding on humans [42]. Currently, in Boyacá, the situation is slightly different because the vector control programs achieved the elimination of the main vector *R. prolixus* in some municipalities [13], but *T. dimidiata* is gaining strength as a vector following the elimination of *R. prolixus* in Boyacá, maintaining the transmission cycle of *T. cruzi* and promoting the circulation of different genotypes of the parasite into the domestic cycle [9]. In Meta (Orinoco region), the situation for *R. prolixus* is the similar to Magdalena, except that there is no evidence of the domiciliation process [43]. Therefore, we can hypothesize that the possible differences the test produce for parasite loads might be due to the ecoepidemiological differences among the species and ecotopes per department and feeding sources as well.

In terms of the results of the parasite loads per species, we found statistically significant differences among *P. geniculatus* with *Ps. arthuri*, *R. colombiensis,* and *R. pallescens* (Figure 3). Studies have found that *P. geniculatus* presents one of the highest rates of *T. cruzi* infection; a wide range of blood meals including humans; and also the presence of many DTUs (TcI-TcV), which is evidence of domiciliation processes due to attraction by artificial light [10]. This species has a long-term defecation time post-feeding (almost an hour after), but what matters is the quantity of parasites they could eject considering their infection rates, as we evidenced here. This characteristic might be important for oral transmission because it has been observed they return to their shelter after feeding and then defecate, probably on food, but no studies have been conducted to determine this [44]. We also found DTU TcI, except from one sample of *P. geniculatus*, which was identified as TcII (Table S2).

*Rhodnius pallescens* is considered a secondary vector that has a wide variety of blood sources, along with other important species such as *P. geniculatus* and *R. prolixus*, and also includes high infection rates (Figure S1). This species is the main vector in Panamá, whose populations are associated with *Attalea butyracea* palms but present sporadic active dispersion towards rural dwellings [45]. Moreover, it has been found with a *T. cruzi* infection rate of 72% in individuals collected inside the houses [45]. In Colombia, *R. pallescens* presents infection rates of 45%, and similar to Panamá, it is a sylvatic species with risk of intrusion in domestic ecotope and it has been involved in oral transmission cases [10,46]. Here, we report a wide range of parasite loads for this species, as far as 9 million parasites (Figure 3 and Figure 4), which can be important for oral outbreaks, as have been hypothesized previously [12,46]. *Rhodnius colombiensis* is another sylvatic triatomine associated with palms (*Attalea butyracea*) that gained epidemiological importance due to its high infection rates with *T. cruzi* and their frequency in invading human dwellings [47]. The parasite loads we found range between 100 and 5000 parasites, which can be important considering its high capacity to transmit *T. cruzi*, specifically TcI sylvatic (TcId) [47]. This DTU is the most frequent found in isolations from oral outbreaks in Colombia [48,49], implying there is an association among the sylvatic transmission cycle that probably can be attributed to secondary vectors [12]. On the other hand, *Psammolestes arthuri* is a sylvatic triatomine that feed on birds and is frequently found in bird nests [50]. It was recently found to be naturally infected with *T. cruzi* with an infection rate of 70%, with mixed infection with many DTUs and feeding on humans with a frequency of 26%, which implies that although its behavior remains ornithophilic, it may be feeding on other animals potentially infected with the parasite and therefore contributing to the maintenance of the parasite’s cycle [8].

The differences observed above might be related to the geographical region, but better sampling is needed to reach a conclusion. The specimens of *P. geniculatus* were collected in some departments with a domestic ecoepidemiological situation (Boyacá, Santander), in contrast to *Ps. arthuri* collected in Casanare, *R. colombiensis* in Tolima, and *R. pallescens* in the northside (Bolivar, Antioquia, Cesar) (Figure 4). *T. dimidiata* could be in a similar situation with the same three sylvatic species mentioned previously. An additional and specific characteristic in this case is that *T. dimidiata* was the second species with the highest and wider range of parasite load behind *P. geniculatus*, even surpassing the main vector *R. prolixus* (Figure 3). This might indicate that the behavior of species with a domestic ecoepidemiological situation is somehow making them become infected with higher loads of parasites, which increase the potential risk of Chagas for humans. This is relevant considering that the interruption of vector-borne transmission can be better in areas where vectors present domiciliated or peri-domiciliated behavior, such as what happened, for instance, in vector control programs for *T. infestans* in Brazil, Chile, Uruguay, Paraguay, and Argentina. In Brazil, the programs have been working since 1975, and by 1993, they were able to report a reduction of infestation by *T. infestans* of 86% [51]. After this stage, most Southern Cone countries entered a phase of surveillance that consisted of monitoring infestation and focal spraying, but nowadays, the challenge is the sustainability of these programs and to deal with the control failures reported in Bolivia and Argentina as a result of triatomines’ resistance to insecticides components [51]. The Southern Cone model was implemented and adapted for the Andean countries and Central America initiatives, where the programs are underway [51].

For the other species evaluated, *T. maculata* in Colombia is considered as secondary, with high percentage of infection of 67% just behind *R. prolixus* and *P. geniculatus,* feeding mainly on human blood [10]. We found a parasitic load slightly higher than that from *T. venosa, R. colombiensis,* and *Ps. arthuri*, but unfortunately, we were only able to analyze two specimens. In Venezuela, it was found that human dwellings could constitute an adequate niche for this species to survive, suggesting a potential domiciliation process [14]. Moreover, along with the results obtained previously, this species’ behavior suggests the existence of new transmission scenarios that together with their presence in the peridomestic ecotope underline the need to evaluate the potential risk this species could have [10]. A study conducted in Brazil evidenced the potential of this species as a vector by evaluating its infestation capacity, short developmental cycle, and defecation time [52]. Once again, this implies the need to start the surveillance of this species once considered sylvatic [7].

*Triatoma dispar* is a sylvatic triatomine found in Antioquia, Cauca, Chocó, Nariño, Valle del Cauca, and Huila [7]. Here, we analyzed two specimens from Chocó that were infected with parasite loads of 55,000 and 7 million parasites, respectively. In Ecuador, it has been found in the peridomicile, which probably could represent an epidemiological risk for *T. cruzi* transmission according to its ecological characteristics [53]. In Colombia, there is no information regarding its ecological behavior, and therefore more studies are needed to unravel the possible importance this species could have in the transmission cycle of the parasite and if it could represent a risk for humans in the future. The results of parasite load of this species were the narrowest and one of the lowest we found. Even though the quantities of parasites we found are not conclusive, it is important to keep studying their importance for *T. cruzi* transmission.

## 5. Conclusions

We highlight that the parasitic loads and DTUs found in species considered secondary must begin to be spotlighted as they might be gaining importance in terms of the parasite’s transmission, mainly due to anthropogenic processes such as deforestation and urbanization that disturb the triatomines ecology and activities that promote the active dispersal of specimens into or near the dwellings [7,10]. Moreover, some of them could represent an imminent risk for oral transmission, as they could reach the dwellings and contaminate food or beverages. Similarly, as happened with *R. colombiensis*, the vectorial capacity for secondary and sylvatic species should be evaluated to determine if this species has a reliable epidemiological relevance for Chagas disease transmission and if there is a correlation between parasite load and vector capacity. Therefore, after *R. prolixus*, the vector control programs in Colombia must focus now on *T. dimidiata* and *T. venosa* and begin the surveillance of other species such as *T. maculata*, *T. dispar,* and *R. pallescens*, considering their parasite load, infection rates, and the importance they have as a vector in other countries of the region such as Venezuela, Panamá, and Brazil.

We had some limitations in our study. For instance, (i) we had an unequal number of samples per species to analyze due to DNA availability (volume) that could have affected the standard deviations in the used statistics, including the lack of sampling effort; (ii) limited geographic representability (only 13 of 31 departments) and sample bias; (iii) we only used one marker for genotyping; and (iv) of the 182 specimens evaluated, only 4 were nymphs. For the standard curve, we only used *R. prolixus*, but we propose our standardized qPCR to evaluate other species and not only adults but more developmental stages of the individuals as well. However, for the DNA extraction, we suggest using only the rectal ampulla and not the whole abdomen section as we did, considering it is where the metacyclic trypomastigotes are present and that the triatomine gut could contain some components that might affect the qPCR reaction [54,55,56]. Additionally, because other infective forms of the parasite might be present in the rectal ampulla, a new approximation based on gene expression specifically for metacyclic trypomastigotes could be implemented. For future studies, we suggest improving the genotyping of *T. cruzi* using more genes and evaluating the presence of other *Trypanosoma* species, which might explain the lack of amplification of some samples in our study. Moreover, even though we provide a close-up of the parasite transmission, we did not evaluate if there was an effective metacyclogenesis, and therefore further studies must include gene markers solely expressed in metacyclic trypomastigotes as an approximation and then evaluate them in the insects’ guts. Lastly, future studies should then estimate the vector capacity and the impact of parasite loads on the transmission.

Our results confirm the suggestions for secondary species and their upcoming relevance for *T. cruzi* transmission in endemic regions for Chagas disease in Colombia. Moreover, we reiterate the need for serological studies to obtain a true picture of the risk the humans are exposed to, as well as vector-specific studies to decipher their potential as *T. cruzi* vectors. To conclude, our parasite load qPCR standardization had great sensitivity, efficiency, and can be useful in future studies directed to contribute with this important issue exposed here. Moreover, we hope this standardized technique can be applied across the country and the region. Additionally, there is still a lack of information about secondary vectors and their ecoepidemiology, and thus we encourage the scientific community to continue deciphering and studying this species with wider sampling and real-time data. Furthermore, we observed that in certain departments where few studies have been conducted, some species could have a huge role for the parasite’s transmission, as is the case for *T. dispar* in Chocó, where the only two individuals we analyzed presented high parasite loads. We also propose not to only focus on the main species such as *R. prolixus* (considering its potential to reinfest and/or recolonize) and *T. dimidiata* (which is gaining importance after *R. prolixus* elimination). We hope the data in the future will be obtained using the methodology exposed here and that they will significantly improve the entomological surveillance and vector control programs, as they could provide more information regarding vector infection and the risk factors that it entails for disease development, especially in CD-endemic areas. The above with the aim to continue understanding the vectors’ behavior and ecology that could lead to a decrease in the CD cases in the Americas, as well as making the vector control programs successful and sustainable.

## Data Availability

All the data is available in the manuscript and the corresponding supplementary files.

## Supplementary Materials

The following supporting information can be downloaded at https://www.mdpi.com/article/10.3390/tropicalmed7120445/s1, Figure S1. Barplot that shows the infection rate per species. Figure S2. Beeswarm plot of the parasites loads per department. Right map shows the different species distribution in Colombia with the same color code of the legend on the left. The color dots in the map do not represent the number of samples in each department. The map was made with QGIS v.3.22. Table S1. Summary table that contains samples’ information for the 182 triatomines analyzed. Table S2. Genotyping result per species. Samples denoted as NA could not be genotyped by conventional PCR.
