# Supplementary material for: Trypanosoma cruzi Parasite Burdens of Several Triatomine Species in Colombia
Source: Trop Med Infect Dis. 2022 Dec 19;7(12):445. doi: 10.3390/tropicalmed7120445 (PMC9782637; doi:10.3390/tropicalmed7120445)
Supplement: Supplementary file 1 [file tropicalmed-07-00445-s001.zip › Table S1.pdf]

Table S1. Summary table that contains samples' information for the 182 triatomines analyzed.

Sample ID	Species	Stage	Country	Department	Municipality	Parasite load	Log10 parasite load	DTU
**35**	*Triatoma dimidiata*	Adult	Colombia	Boyacá	Soatá	35,397,384	7.5489712	TcI
**63**	*Triatoma dimidiata*	Adult	Colombia	Boyacá	Soatá	85,356,448	7.9312363	TcI
**75**	*Triatoma dimidiata*	Adult	Colombia	Boyacá	Tipacoque	14,829,605	7.1711296	TcI
**84**	*Triatoma dimidiata*	Adult	Colombia	Boyacá	Tipacoque	61,558,340	7.7892869	TcI
**87**	*Triatoma dimidiata*	Adult	Colombia	Boyacá	Soatá	1,157,104,320	9.0633725	TcI
**94**	*Triatoma dimidiata*	Adult	Colombia	Boyacá	Soatá	2,311	3.3637999	TcI
**101**	*Triatoma dimidiata*	Nymph	Colombia	Boyacá	Tipacoque	324	2.5105450	TcI
**113**	*Triatoma dimidiata*	Adult	Colombia	Boyacá	Soatá	1,461,363,200	9.1647582	TcI
**121**	*Triatoma dimidiata*	Adult	Colombia	Boyacá	Soatá	9,165,565	6.9621592	TcI
**122**	*Triatoma dimidiata*	Nymph	Colombia	Boyacá	Soatá	8,663	3.9376683	TcI
**124**	*Triatoma dimidiata*	Adult	Colombia	Boyacá	Tipacoque	715	2.8543060	TcI
**132**	*Triatoma dimidiata*	Adult	Colombia	Boyacá	Soatá	271,009	5.4329837	TcI
**134**	*Triatoma dimidiata*	Adult	Colombia	Boyacá	Soatá	24,664,522	7.3920727	TcI
**157**	*Triatoma dimidiata*	Adult	Colombia	Boyacá	Socota	12,094	4.0825700	TcI
**159**	*Triatoma dimidiata*	Adult	Colombia	Boyacá	Soatá	52,042,616	7.7163591	TcI
**160**	*Triatoma dimidiata*	Adult	Colombia	Boyacá	Boavita	10,906	4.0376655	TcI
**167**	*Triatoma dimidiata*	Adult	Colombia	Boyacá	Susacón	5,902	3.7709992	TcI
**172**	*Triatoma dimidiata*	Adult	Colombia	Boyacá	Soatá	878,414	5.9436992	TcI
**173**	*Triatoma dimidiata*	Adult	Colombia	Boyacá	Socota	30,407,030	7.4829740	TcI
**186**	*Triatoma dimidiata*	Adult	Colombia	Boyacá	Socota	2,129,457	6.3282689	TcI
**196**	*Triatoma dimidiata*	Adult	Colombia	Boyacá	Soatá	5,262,563	6.7211973	TcI
**203**	*Triatoma dimidiata*	Adult	Colombia	Boyacá	Boavita	906	2.9571282	TcI
**215**	*Triatoma dimidiata*	Adult	Colombia	Boyacá	Soatá	35,752,096	7.5533015	TcI
**221**	*Triatoma dimidiata*	Nymph	Colombia	Boyacá	Tipacoque	15,968	4.2032505	TcI

**235**	*Triatoma dimidiata*	Adult	Colombia	Boyacá	Tipacoque	42,480	4.6281845	TcI
**243**	*Triatoma dimidiata*	Adult	Colombia	Boyacá	Socota	121,577,568	8.0848535	TcI
**244**	*Triatoma dimidiata*	Nymph	Colombia	Boyacá	Panqueba	19,033,480	7.2795182	TcI
**247**	*Triatoma dimidiata*	Adult	Colombia	Boyacá	San Mateo	48,741,376	7.6878978	TcI
**253**	*Triatoma dimidiata*	Adult	Colombia	Boyacá	Soatá	30,008,396	7.4772428	TcI
**265**	*Triatoma dimidiata*	Adult	Colombia	Boyacá	Socota	60,196	4.7795676	TcI
**DNA 132d**	*Rhodnius prolixus*	Adult	Colombia	Casanare	Paz de Ariporo	-	-	-
**DNA 133d**	*Rhodnius prolixus*	Adult	Colombia	Casanare	Paz de Ariporo	31,704,430	7.5011199	TcI
**DNA 134d**	*Rhodnius prolixus*	Adult	Colombia	Casanare	Paz de Ariporo	-	-	-
**DNA 136**	*Rhodnius pallescens*	Adult	Colombia	Cesar	Valledupar	50,984	4.7074371	TcI
**DNA 137**	*Rhodnius pallescens*	Adult	Colombia	Cesar	Valledupar	-	-	-
**DNA 138**	*Rhodnius pallescens*	Adult	Colombia	Cesar	Valledupar	-	-	-
**DNA 139**	*Rhodnius pallescens*	Adult	Colombia	Cesar	Valledupar	74,601	4.8727455	TcI
**DNA 140d**	*Rhodnius prolixus*	Adult	Colombia	Arauca	Puerto Rondón	26,070	4.4161442	TcI
**DNA 141d**	*Rhodnius prolixus*	Adult	Colombia	Arauca	Puerto Rondón	787	2.8960731	NA
**DNA 144**	*Rhodnius pallescens*	Adult	Colombia	Antioquia	Necocli	27,816	4.4442991	TcI
**DNA 145**	*Rhodnius pallescens*	Adult	Colombia	Antioquia	Necocli	787	2.8960527	NA
**DNA 146**	*Rhodnius pallescens*	Adult	Colombia	Antioquia	Necocli	9,719,022	6.9876226	TcI
**DNA 147**	*Rhodnius pallescens*	Adult	Colombia	Antioquia	Necocli	1,089,328	6.0371585	TcI
**DNA 147d**	*Rhodnius prolixus*	Adult	Colombia	Casanare	Tamara	2,332,787	6.3678750	TcI
**DNA 152d**	*Rhodnius prolixus*	Adult	Colombia	Casanare	Tamara	5,442,715	6.7358156	TcI

**DNA 154d**	*Rhodnius prolixus*	Adult	Colombia	Casanare	Tamara	39,216,532	7.5934692	TcI
**DNA 161d**	*Rhodnius prolixus*	Adult	Colombia	Casanare	Pore	362,335	5.5591104	TcI
**DNA 162d**	*Rhodnius prolixus*	Adult	Colombia	Casanare	Pore	3,907,144	6.5918594	TcI
**DNA 167d**	*Rhodnius prolixus*	Adult	Colombia	Casanare	Pore	22,351,330	7.3493034	TcI
**DNA 169d**	*Rhodnius prolixus*	Adult	Colombia	Casanare	Pore	36,838,908	7.5663067	TcI
**DNA 185**	*Rhodnius pallescens*	Adult	Colombia	Bolivar	Mompox	16,066	4.2058951	-
**DNA 186**	*Rhodnius pallescens*	Adult	Colombia	Bolivar	Mompox	16,066	4.2058951	NA
**DNA 187**	*Rhodnius pallescens*	Adult	Colombia	Bolivar	Mompox	627	2.7970192	NA
**DNA 188**	*Rhodnius pallescens*	Adult	Colombia	Bolivar	Mompox	336	2.5260656	NA
**DNA 201d**	*Panstrongylus geniculatus*	Adult	Colombia	Meta	Restrepo	10,291,462	7.0124771	TcI
**DNA 202d**	*Panstrongylus geniculatus*	Adult	Colombia	Meta	Restrepo	162,278	5.2102590	TcI
**DNA 203d**	*Panstrongylus geniculatus*	Adult	Colombia	Meta	Restrepo	39,889	4.6008550	TcI
**DNA 204d**	*Panstrongylus geniculatus*	Adult	Colombia	Meta	Restrepo	149,983,808	8.1760444	TcI
**DNA 207d**	*Panstrongylus geniculatus*	Adult	Colombia	Meta	Restrepo	18,781,748	7.2737360	NA
**DNA 208d**	*Panstrongylus geniculatus*	Adult	Colombia	Meta	Restrepo	73,967	4.8690389	TcI
**DNA 209d**	*Panstrongylus geniculatus*	Adult	Colombia	Meta	Restrepo	25,790,952	7.4114674	TcI
**DNA 212d**	*Panstrongylus geniculatus*	Adult	Colombia	Meta	Restrepo	159,226,192	8.2020145	NA

**DNA 213d**	*Panstrongylus geniculatus*	Adult	Colombia	Meta	Restrepo	8,173,628	6.9124148	TcI
**DNA 214d**	*Panstrongylus geniculatus*	Adult	Colombia	Meta	Restrepo	5,727,317	6.7579512	TcII
**DNA 215d**	*Panstrongylus geniculatus*	Adult	Colombia	Meta	Restrepo	10,112,290	7.0048495	TcI
**DNA 230**	*Psammolestes arthuri*	Adult	Colombia	Casanare	Mani	68	1.8356851	NA
**DNA 383**	*Rhodnius colombiensis*	Adult	Colombia	Tolima	Coyaima	585	2.7672935	NA
**DNA 384**	*Rhodnius colombiensis*	Adult	Colombia	Tolima	Coyaima	472	2.6738620	NA
**DNA 385**	*Rhodnius colombiensis*	Adult	Colombia	Tolima	Coyaima	-	-	-
**DNA 386**	*Rhodnius colombiensis*	Adult	Colombia	Tolima	Coyaima	260	2.4145308	NA
**DNA 387**	*Rhodnius colombiensis*	Adult	Colombia	Tolima	Coyaima	2,759	3.4407426	NA
**DNA 398**	*Rhodnius colombiensis*	Adult	Colombia	Tolima	Coyaima	2,429	3.3853937	NA
**DNA 399**	*Rhodnius colombiensis*	Adult	Colombia	Tolima	Coyaima	266	2.4242862	NA
**DNA 400**	*Rhodnius colombiensis*	Adult	Colombia	Tolima	Coyaima	261	2.4166632	NA
**DNA 401**	*Rhodnius colombiensis*	Adult	Colombia	Tolima	Coyaima	111	2.0463024	NA
**DNA 402**	*Rhodnius colombiensis*	Adult	Colombia	Tolima	Coyaima	-	-	-
**DNA 425**	*Rhodnius pallescens*	Adult	Colombia	Bolivar	San Fernando	138	2.1390807	NA
**DNA 426**	*Rhodnius pallescens*	Adult	Colombia	Bolivar	San Fernando	-	-	-
**DNA 427**	*Rhodnius pallescens*	Adult	Colombia	Bolivar	San Fernando	34	1.5269295	NA

**DNA 428**	*Rhodnius pallescens*	Adult	Colombia	Bolivar	San Fernando	119	2.0749502	NA
**DNA105d**	*Rhodnius prolixus*	Adult	Colombia	Arauca	Tame	1,095	3.0393868	NA
**DNA112d**	*Rhodnius prolixus*	Adult	Colombia	Arauca	Saravena	169	2.2269245	NA
**DNA115d**	*Rhodnius prolixus*	Adult	Colombia	Arauca	Saravena	526,503	5.7214005	TcI
**DNA117d**	*Rhodnius prolixus*	Adult	Colombia	Arauca	Arauca	529	2.7234248	TcI
**DNA118d**	*Rhodnius prolixus*	Adult	Colombia	Arauca	Arauca	12,895	4.1104207	TcI
**DNA12d**	*Rhodnius pallescens*	Adult	Colombia	Bolivar	Mompox	32	1.4984311	-
**DNA14d**	*Rhodnius pallescens*	Adult	Colombia	Bolivar	Mompox	-	-	-
**DNA15d**	*Rhodnius pallescens*	Adult	Colombia	Bolivar	Mompox	21	1.3173026	NA
**DNA19d**	*Rhodnius prolixus*	Adult	Colombia	Magdalena	Sierra Nevada de Santa Marta	3,640	3.5610633	-
**DNA1d**	*Eratyrus mucronatus*	Adult	Colombia	Norte de santander	Los patios (Santa clara)	302	2.4801783	TcI
**DNA21d**	*Rhodnius prolixus*	Adult	Colombia	Magdalena	Sierra Nevada de Santa Marta	1,768	3.2474050	-
**DNA251d**	*Panstrongylus geniculatus*	Adult	Colombia	Casanare	Paz de Ariporo	1,409,156	6.1489590	TcI
**DNA253d**	*Panstrongylus geniculatus*	Adult	Colombia	Casanare	Paz de Ariporo	7,949,679	6.9003496	TcI
**DNA255d**	*Psammolestes arthuri*	Adult	Colombia	Casanare	Pore	14	1.1604392	NA
**DNA257d**	*Psammolestes arthuri*	Adult	Colombia	Casanare	Pore	-	-	-
**DNA261d**	*Psammolestes arthuri*	Adult	Colombia	Casanare	Pore	66	1.8200522	NA
**DNA263d**	*Psammolestes arthuri*	Adult	Colombia	Casanare	Pore	25	1.4006630	NA
**DNA285d**	*Psammolestes arthuri*	Adult	Colombia	Casanare	Mani	1,016	3.0066914	NA

**DNA299d**	*Psammolestes arthuri*	Adult	Colombia	Casanare	Mani	35	1.5378319	NA
**DNA2d**	*Eratyrus mucronatus*	Adult	Colombia	Norte de santander	Los patios (Santa clara)	628	2.7978026	NA
**DNA301d**	*Psammolestes arthuri*	Adult	Colombia	Casanare	Mani	4,402	3.6436879	NA
**DNA305d**	*Psammolestes arthuri*	Adult	Colombia	Casanare	Mani	-	-	-
**DNA307d**	*Psammolestes arthuri*	Adult	Colombia	Casanare	Mani	-	-	-
**DNA309d**	*Psammolestes arthuri*	Adult	Colombia	Casanare	Mani	-	-	-
**DNA315d**	*Panstrongylus geniculatus*	Adult	Colombia	Casanare	Paz de Ariporo	-	-	-
**DNA319d**	*Rhodnius prolixus*	Adult	Colombia	Arauca	Fortul	52,900,404	7.7234590	TcI
**DNA323d**	*Rhodnius prolixus*	Adult	Colombia	Arauca	Fortul	12,528	4.0978742	TcI
**DNA325d**	*Rhodnius prolixus*	Adult	Colombia	Arauca	Fortul	18,831	4.2748626	TcI
**DNA329d**	*Rhodnius prolixus*	Adult	Colombia	Arauca	Puerto Rondón	-	-	-
**DNA32d**	*Rhodnius prolixus*	Adult	Colombia	Magdalena	Sierra Nevada de Santa Marta	153	2.1835157	NA
**DNA333d**	*Panstrongylus geniculatus*	Adult	Colombia	Arauca	Arauca	-	-	-
**DNA341d**	*Panstrongylus geniculatus*	Adult	Colombia	Arauca	Fortul	-	-	-
**DNA349d**	*Triatoma maculata*	Adult	Colombia	Guajira		482	2.6826938	NA
**DNA34d**	*Rhodnius prolixus*	Adult	Colombia	Magdalena	Sierra Nevada de Santa Marta	-	-	-
**DNA371d**	*Panstrongylus geniculatus*	Adult	Colombia	Santander	Playon	-	-	-
**DNA377d**	*Psammolestes arthuri*	Adult	Colombia	Casanare	Tamara	2,391	3.3786480	TcI

**DNA379d**	*Psammolestes arthuri*	Adult	Colombia	Casanare	Tamara	-	-	-
**DNA381d**	*Psammolestes arthuri*	Adult	Colombia	Casanare	Tamara	-	-	-
**DNA383d**	*Psammolestes arthuri*	Adult	Colombia	Casanare	Tamara	-	-	-
**DNA385d**	*Psammolestes arthuri*	Adult	Colombia	Casanare	Tamara	-	-	-
**DNA387d**	*Psammolestes arthuri*	Adult	Colombia	Casanare	Tamara	4,681	3.6703173	TcI
**DNA38d**	*Rhodnius prolixus*	Adult	Colombia	Magdalena	Von	-	-	-
**DNA391d**	*Psammolestes arthuri*	Adult	Colombia	Casanare	Tamara	-	-	-
**DNA395d**	*Psammolestes arthuri*	Adult	Colombia	Casanare	Tamara	375	2.5743717	NA
**DNA397d**	*Psammolestes arthuri*	Adult	Colombia	Casanare	Monterrey	-	-	-
**DNA399d**	*Psammolestes arthuri*	Adult	Colombia	Casanare	Monterrey	1,040	3.0168846	NA
**DNA3d**	*Eratyrus mucronatus*	Adult	Colombia	Norte de santander	Los patios (Santa clara)	91,116	4.9595957	TcI
**DNA403d**	*Psammolestes arthuri*	Adult	Colombia	Casanare	Monterrey	-	-	-
**DNA405d**	*Psammolestes arthuri*	Adult	Colombia	Casanare	Monterrey	661,689	5.8206537	NA
**DNA409d**	*Triatoma maculata*	Adult	Colombia	Casanare	Paz de Ariporo	51,683	4.7133506	TcI
**DNA413d**	*Psammolestes arthuri*	Adult	Colombia	Casanare	Monterrey	-	-	-
**DNA415d**	*Psammolestes arthuri*	Adult	Colombia	Casanare	Monterrey	4,927	3.6925973	NA

**DNA42**	*Rhodnius colombiensis*	Adult	Colombia	Tolima	Coyaima	2,276	3.3570795	NA
**DNA45d**	*Rhodnius prolixus*	Adult	Colombia	Magdalena	Von	715	2.8541056	NA
**DNA462d**	*Panstrongylus geniculatus*	Adult	Colombia	Boyacá	Susacón	3,434	3.5358364	NA
**DNA464d**	*Panstrongylus geniculatus*	Adult	Colombia	Boyacá	Susacón	-	-	-
**DNA466d**	*Panstrongylus geniculatus*	Adult	Colombia	Boyacá	Soata	2,628,739	6.4197474	TcI
**DNA468d**	*Panstrongylus geniculatus*	Adult	Colombia	Boyacá	Soata	9,636,328	6.9839116	TcI
**DNA470d**	*Panstrongylus geniculatus*	Adult	Colombia	Boyacá	San Pablo de Borbur	206,605	5.3151416	TcI
**DNA476d**	*Triatoma venosa*	Adult	Colombia	Casanare	Garoa	-	-	-
**DNA478d**	*Triatoma venosa*	Adult	Colombia	Casanare	Garoa	1,667	3.2218084	NA
**DNA47d**	*Rhodnius prolixus*	Adult	Colombia	Magdalena	Von	410	2.6123279	NA
**DNA480d**	*Rhodnius prolixus*	Adult	Colombia	Boyacá	Cubará	1,333	3.1248256	NA
**DNA484d**	*Rhodnius prolixus*	Adult	Colombia	Boyacá	Cubará	808	2.9072037	NA
**DNA488d**	*Triatoma venosa*	Adult	Colombia	Casanare	Garoa	-	-	-
**DNA48d**	*Rhodnius prolixus*	Adult	Colombia	Magdalena	Von	682	2.8340398	NA
**DNA490d**	*Triatoma venosa*	Adult	Colombia	Casanare	Garoa	-	-	-
**DNA492d**	*Triatoma venosa*	Adult	Colombia	Boyacá	Pachavita	6,708	3.8266168	NA
**DNA4d**	*Eratyrus mucronatus*	Adult	Colombia	Norte de santander	San cayetano (La playa)	93,915	4.9727336	TcI
**DNA64**	*Rhodnius colombiensis*	Adult	Colombia	Tolima	Coyaima	4,902	3.6903502	TcI
**DNA65d**	*Rhodnius prolixus*	Adult	Colombia	Arauca	Tame	-	-	-
**DNA67**	*Rhodnius colombiensis*	Adult	Colombia	Tolima	Coyaima	302	2.4805168	NA
**DNA71**	*Rhodnius prolixus*	Adult	Colombia	Antioquia	Necocli	329	2.5171222	NA

**DNA72**	*Rhodnius prolixus*	Adult	Colombia	Antioquia	Necocli	200	2.3018741	NA
**DNA80d**	*Panstrongylus geniculatus*	Adult	Colombia	Santander	Mogotes	28,438,366	7.4539046	TcI
**DNA94d**	*Rhodnius prolixus*	Adult	Colombia	Casanare	Yopal	108,849	5.0368248	TcI
**DNA95d**	*Rhodnius prolixus*	Adult	Colombia	Casanare	Yopal	2,242,750	6.3507808	TcI
**DNA96d**	*Rhodnius prolixus*	Adult	Colombia	Casanare	San Luis de Palenque	62,999	4.7993333	TcI
**Tdis1**	*Triatoma dispar*	Adult	Colombia	Chocó	San José del Palmar	7,609,587	6.8813611	NA
**Tdis2**	*Triatoma dispar*	Adult	Colombia	Chocó	San José del Palmar	55,199	4.7419312	TcI
**Tv1**	*Triatoma venosa*	Adult	Colombia	Boyacá	Zetaquira	147,299	5.1682012	NA
**Tv10**	*Triatoma venosa*	Adult	Colombia	Boyacá	Garagoa	1,676	3.2242100	NA
**Tv11**	*Triatoma venosa*	Adult	Colombia	Boyacá	Guateque	287	2.4580768	NA
**Tv12**	*Triatoma venosa*	Adult	Colombia	Boyacá	Guateque	513	2.7104995	TcI
**Tv13**	*Triatoma venosa*	Adult	Colombia	Boyacá	Zetaquira	1,031	3.0132632	NA
**Tv14**	*Triatoma venosa*	Adult	Colombia	Boyacá	MIraflores	2,227	3.3476923	NA
**Tv15**	*Triatoma venosa*	Adult	Colombia	Boyacá	Chinavita	417	2.6204101	NA
**Tv16**	*Triatoma venosa*	Adult	Colombia	Boyacá	Chinavita	465	2.6675208	NA
**Tv17**	*Triatoma venosa*	Adult	Colombia	Boyacá	Garagoa	115	2.0591517	NA
**Tv18**	*Triatoma venosa*	Adult	Colombia	Boyacá	Garagoa	124	2.0924832	NA
**Tv19**	*Triatoma venosa*	Adult	Colombia	Boyacá	Garagoa	1,305	3.1155790	NA
**Tv2**	*Triatoma venosa*	Adult	Colombia	Boyacá	Garagoa	88,358	4.9462467	TcI
**Tv20**	*Triatoma venosa*	Adult	Colombia	Boyacá	Miraflores	1,052	3.0219047	NA
**Tv21**	*Triatoma venosa*	Adult	Colombia	Boyacá	Zetaquira	417	2.6196652	NA
**Tv22**	*Triatoma venosa*	Adult	Colombia	Boyacá	Chinavita	23	1.3710638	NA
**Tv3**	*Triatoma venosa*	Adult	Colombia	Boyacá	Zetaquira	1,385	3.1415686	NA
**Tv4**	*Triatoma venosa*	Adult	Colombia	Boyacá	Miraflores	7,117	3.8523257	NA
**Tv5**	*Triatoma venosa*	Adult	Colombia	Boyacá	Miraflores	1,930	3.2855716	NA
**Tv6**	*Triatoma venosa*	Adult	Colombia	Boyacá	Guateque	4,750,815	6.6767681	TcI

**Tv7**	*Triatoma venosa*	Adult	Colombia	Boyacá	Zetaquira	2,204	3.3431311	TcI
**Tv8**	*Triatoma venosa*	Adult	Colombia	Boyacá	La Capilla	393	2.5944049	NA
**Tv9**	*Triatoma venosa*	Adult	Colombia	Boyacá	Somondoco	739	2.8687955	NA
