# Supplementary material for: Trypanosoma cruzi Parasite Burdens of Several Triatomine Species in Colombia
Source: Trop Med Infect Dis. 2022 Dec 19;7(12):445. doi: 10.3390/tropicalmed7120445 (PMC9782637; doi:10.3390/tropicalmed7120445)
Supplement: Supplementary file 1 [file tropicalmed-07-00445-s001.zip › Table S2.pdf]

Table S2. Genotyping result per species. Samples denoted as NA could not be genotyped by conventional PCR.

| **Species** | **NA** | **TcI** | **TcII** |
|---|---|---|---|
| *Eratyrus mucronatus* | 1 | 3 | - |
| *Panstrongylus geniculatus* | 3 | 14 | 1 |
| *Psammolestes arthuri* | 11 | 2 | - |
| *Rhodnius colombiensis* | 10 | 1 | - |
| *Rhodnius pallescens* | 8 | 5 | - |
| *Rhodnius prolixus* | 13 | 18 | - |
| *Triatoma dimidiata* | - | 30 | - |
| *Triatoma dispar* | 1 | 1 | - |
| *Triatoma maculata* | 1 | 1 | - |
| *Triatoma venosa* | 20 | 4 | - |
| **Total** | **66** | **79** | **1** |
